# A cross-sectional study on oral health and dental care in intellectually able adults with autism spectrum disorder

**DOI:** 10.1186/s12903-015-0065-z

**Published:** 2015-07-15

**Authors:** My Blomqvist, Susanne Bejerot, Göran Dahllöf

**Affiliations:** Division of Paediatric Dentistry, Department of Dental Medicine, Karolinska Institutet, P.O. Box 4064, SE-14104 Huddinge, Sweden; Department of Clinical Neuroscience, Karolinska Institutet, Tomtebodavägen 18A, SE-171 77 Stockholm, Sweden; Department of Psychiatry, Örebro University, Örebro, Sweden

**Keywords:** Autism, Asperger syndrome, Adult, Dental caries, Dental appointments

## Abstract

**Background:**

Autism spectrum disorder (ASD) is characterized by impairments in social interaction and communication, restricted patterns of behaviour, and unusual sensory sensitivities. The hypotheses to be tested were that adult patients with ASD have a higher caries prevalence, have more risk factors for caries development, and utilize dental health care to a lesser extent than people recruited from the normal population.

**Methods:**

Forty-seven adults with ASD, (25 men, 22 women, mean age 33 years) and of normal intelligence and 69 age- and sex-matched typical controls completed a dental examination and questionnaires on oral health, dental hygiene, dietary habits and previous contacts with dental care.

**Results:**

Except for increased number of buccal gingival recessions, the oral health was comparable in adults with ASD and the control group. The group with ASD had less snacking, but also less frequent brushing of teeth in the mornings. The stimulated saliva secretion was lower in the ASD group, regardless of medication. Frequencies of dental care contacts were equal in both groups. The most common reason for missing a dental appointment was forgetfulness in the ASD group.

**Conclusions:**

Adults with ASD exhibited more gingival recessions and considerably lower saliva flow compared to healthy controls. Despite equal caries prevalence, the risk for reduced oral health due to decreased salivary flow should be taken into consideration when planning dental care for patients with ASD. Written reminders of dental appointments and written and verbal report on oral health status and oral hygiene instructions are recommended.

## Background

Autism spectrum disorder (ASD) is a persistent neurodevelopmental condition with early childhood onset. The prevalence of ASD seems to be rising and was recently estimated at 1.7 % in the UK [1]. ASD is characterized by impairments in social interaction and communication, and restricted, repetitive patterns of behaviour, interests or activities and unusual sensory interests or sensitivities [2]. In the recently revised American classification system for psychiatric disorders, the DSM-5 [2], ASD now includes previously separate diagnostic categories such as autistic disorder, Asperger disorder, and pervasive developmental disorder-not otherwise specified, specified (PDD-NOS) in the DSM-IV-TR [3].

People with ASD are impaired in building flexible predictions and expectations, which is very much needed in the highly unpredictable social world [4]. Although sensory abnormalities to a greater extent affect those with more severe autistic traits compared to those with less autistic traits [5], hyper-perception is consistently observed in people with ASD [6]. The combination of a hyper-perception and an inability to anticipate sensory inputs may contribute to difficulties regarding dental care, as dental care often includes bright light, loud noises and strong tastes or smells. Accordingly, sensory sensitivities in children with ASD have shown to be related to behaviour difficulties in the dental office [7].

Studies on oral health in patients with ASD have conflicting results. In a population-based sample of autistic children, parents reported poorer condition of their children’s teeth compared to other children [8]. On the other hand, other studies report similar caries prevalence in children with ASD and controls [9, 10] or lower caries prevalence and severity [11, 12]. Pharmacological management of patients with ASD often includes medications affecting salivary flow, such as treatments for mood disorders, attention deficit, aggression, anxiety and insomnia, which might contribute to an increased caries risk [13]. The few studies published describing utilization of dental services in patients with ASD, have focused on barriers to access dental care [14] and difficulties to find a dentist with the skills or willingness to work with patients with disabilities [15]. Not finding the right dentist was in fact the most frequent reason cited for not having a regular dental provider [15]. To our knowledge, there are yet no studies on utilization of dental services in adults with ASD.

The aim of this study was to investigate the oral health, oral health behaviour, and contacts with dental care in adults with ASD compared to a control group. The hypotheses to be tested in this study were that adult patients with ASD have a higher caries prevalence, have more risk factors for caries development and utilize dental health care to a lesser extent than people recruited from the normal population.

## Methods

### Study setting

This study is a cross-sectional study, in which a group of adults diagnosed with ASD is compared to a group of typical adults, living in the northern Stockholm area. All participants attended a dental examination appointment and completed questionnaires. After the examination, each participant was provided a written and verbal report on his/her own dental status and what dental treatment was recommended. Written consent was obtained from the participants, and ethical approval was obtained from the regional ethical board in Stockholm, Sweden (2008/874-31/4).

### Participants

The participants with ASD were recruited from a tertiary psychiatric unit for diagnosing ASD in adulthood, the Neuropsychiatric unit, Northern Stockholm psychiatric clinic, and from a community-based unit for adults with ASD located in Stockholm, Sweden. They had all been diagnosed with ASD prior to this study. Local diagnostic procedures require that a senior psychiatrist and a psychologist, both trained in diagnosing ASD, perform the assessments which take 12–18 h to complete, during a time period of two weeks to three months. In order to obtain an early developmental history supporting ASD an interview with a parent is mandatory. The diagnosis of ASD is established in consensus between the psychiatrist and psychologist. The assessment includes Wechsler Adult Intelligence Scale (in order to identify individuals with an intellectual disability) and other neuropsychological tests, and structured and semi-structured clinical interviews following the DSM-IV-TR criteria [3] for autistic disorder, Asperger disorder or PDD-NOS.

The participants with ASD were first contacted by means of an invitation letter. An invitation letter was sent to 62 eligible patients diagnosed with ASD at the Northern Stockholm psychiatric clinic and to a large cohort consisting of all subjects with ASD without co-existing intellectual disability, registered at the community-based unit for adults with ASD in Stockholm county (n = 339). Sixty-nine patients with ASD agreed to participate by a letter of reply; fifty-nine of these completed a telephone interview. Seven patients were unavailable for the dental appointment and another five failed to attend, resulting in a final study group comprising of 47 adults with ASD (25 males, 22 females).

Sixty-nine (34 males, 35 females) typical adults were recruited from six dental clinics in the Stockholm area. The control group was matched to the clinical group with regard to age, gender and area of residence, as area of residence in Stockholm closely reflects socioeconomic background.

Exclusion criteria for all participants were diagnosis of intellectual disability, a history of brain damage, current or past neurological disorder, epilepsy, alcohol abuse, or dependence, past or present substance abuse and psychosis. Their medical records were reviewed in order to identify exclusion criteria. In addition, scores above cut-off for a probable ASD, according to Autism-Spectrum Quotient (AQ), a rating scale for assessing autistic traits (described below), was an exclusion criterion for the controls.

### Demographic and background information

Demographic and background information was collected from the participants in conjunction with the dental appointment, using a self-administered questionnaire comprising of questions on medical diagnosis, medication, country of birth of parents, educational level, occupational status and tobacco use (Table 1).

**Table 1:** Socio-demographic characteristics of adults with autism spectrum disorder (*n* = 47) and a control group (*n* = 69)

	ASD group	Control group	
	*n* = 47	*n* = 69	*p*-value
Mean age (years)	33 ± 8	34 ± 7	0.466^a^
Male sex n (%)	25 (53)	34 (49)	0.679^b^
Mother’s country of birth, n (%)			
Sweden	39 (83)	57 (83)	
Other Nordic countries	4 (9)	5 (7)	
Other European countries	1 (2)	2 (3)	
Rest of the world	3 (6)	5 (7)	0.985^b^
Father’s country of birth, n (%)			
Sweden	36 (77)	59 (86)	
Other Nordic countries	5 (11)	3 (4)	
Other European countries	3 (6)	2 (3)	
Rest of the world	3 (6)	5 (7)	0.441^b^
Highest educational level, n (%)			
Elementary school	3 (6)	2 (3)	
High school	28 (60)	25 (36)	
University/college	16 (34)	41 (59)	0.021^b^
Working or studying, n (%)			
Full time	11 (23)	63 (91)	
Part time	7 (15)	5 (7)	
Unemployed/not studying	29 (62)	1 (1)	<0.001^b^
Smoking, n (%)	2/47 (4)	9/69 (13)	0.195^c^
Snuff, n (%)	4/47 (9)	8/69 (12)	0.759^c^
Medication, n (%)	31/47 (66)	7/69 (10)	<0.001^b^
Medication with reported side-effect hyposalivation, n (%)	23/47 (47)	1/69 (1)	<0.001^b^
AQ single count (range)^d^	28.7 ± 10.4 (5.0-46.0)	11.4 ± 4.5 (4.0-28.0)	p < 0.001^a^

*ASD* autism spectrum disorder, *AQ* Autism-spectrum quotient

^a^t-test

^b^Chi2 test

^c^Fisher’s exact test

^d^data missing for one individual in the ASD group and one individual in the control group

### Screening for ASD

In order to screen for behaviours related to ASD, all participants completed the Autism-Spectrum Quotient, AQ [16]. The AQ is a widely used self-rating scale that describes a variety of traits typically observed in individuals with ASD and is designed to be used in adults with ASD and of at least average intelligence [17]. It consists of 50 items, assessing personal preferences and habits. Subjects rate to what extent they agree or disagree with the statements on a 4-point Likert-type scale, ranging from definitely agree (0) to definitely disagree (3). Items are subsequently coded dichotomously into 0 and 1 to reflect the absence or presence of each symptom Total AQ scores reflect the sum of all items; the lowest possible score (i.e., 0) indicates no autistic traits and the highest possible score (50) indicates severe autistic traits. A score above 32 has been proposed as a cut off score for probable ASD [16], but people with a confirmed ASD diagnosis sometimes have lower scores [18]. Severity of ASD is reflected through higher scores on the AQ [19].

### Dental examination

All participants underwent a clinical examination in the dental chair, and two bite-wing radiographs were taken. The researcher who examined the patients in the ASD group (MB), was not previously known to the participants. The patients in the control group were examined by their general dentist. The number of permanent teeth was registered. Manifest dental caries was scored [20], and non-cavitated lesions on smooth surfaces, defined as white chalky areas, were rated separately as initial caries lesions. One examiner (XX) assessed manifest and initial interdental lesions on radiographs from all patients [21]. The frequency of gingival sites that exhibited bleeding on probing, the gingival bleeding index (GBI%), was calculated [22]. Gingival recessions on the buccal aspect of all teeth were registered as the distance from the cemento-enamel junction to the marginal gingiva in millimetres (mm). Occurrence or absence of supragingival calculus was registered. Paraffin-stimulated whole saliva was collected over five minutes and salivary secretion rate was determined. Manifest and enamel caries lesions were registered on bite-wing radiographs, All radiograhs were read by one examiner to in part control for the possible bias of other examiners in the control group.

Regarding bite-wing radiographs assessment of intraexaminer agreement was tested and retested in 30 randomly selected cases with a 2-month interval. Exact agreement was found for 99 % of the comparisons regarding manifest lesions and 99 % regarding initial caries lesions. Weighted kappa was 0.99 regarding manifest lesions and 0.99 regarding initial caries lesions.

### Estimation of oral health

In a self-administered questionnaire, all participants were asked to estimate their oral health (very good/good/bad/very bad). The question has previously been used in a Norwegian study on dental anxiety, but the instrument is not properly validated [23]. In the statistical analysis the answers “very good/good” were combined into one group, versus the answers “bad/very bad”.

### Dental hygiene and dietary habits

The patients completed one questionnaire on oral health and dental hygiene habits and one on dietary habits. The oral health and dental hygiene habits questionnaire comprised seven questions on oral hygiene habits, use of fluoride, and self-perceived gingival bleeding. The frequency of each habit was expressed in five alternatives: never/once a week/2–3 times a week/4–6 times a week/every day. In the statistical analyses, the categories once a week/2–3 times a week/4–6 times a week were combined into one group “not every day”. These questions have previously been used in Swedish studies but are not as yet validated [24–26]. The dietary habits questionnaire comprised three questions on frequency of food and beverage intake and frequency of fermentable carbohydrate snacking. Each question consisted of the response alternatives yes or no. The questions were validated in a clinical setting in the United States and include the dietary behaviours that were most predictive of caries risk [27]. The questions have previously been used in Swedish studies [25, 26].

### Dental experience

All participants completed a questionnaire on previous dental experience. They were asked if they had visited the dentist after the age of 20 years (no/yes; dental appointments are free of charge in Sweden until age 20), if they had cancelled or missed any appointments during the last two years (no/once/more than once/not relevant) and reason for the cancellation or missed appointment. The questions have previously been used in a Norwegian study on dental anxiety, but have not been validated [23]. Responses reflecting “once” and “more than once” were combined into one group “yes”, versus “no” in the statistical analysis.

### Statistical analysis

Mean scores were calculated for the AQ. Missing data were analysed by multiplying the mean item score for each response by the number of items in each scale, thus obtaining comparable scores. This method was chosen after it had been ascertained that no statistically significant differences occurred between the overall mean scores calculated in this way and the overall mean scores obtained using only complete questionnaires. A response rate above 80 % in AQ was required for the questionnaire to be included.

Student’s t-test was used to determine the significance of differences between continuous variables for the two groups, except for calculations on subgroups with no medication where Mann Whitney U-test was used. Categorical data were compared using the chi-square test. If the observed frequency was below five in one cell, Fisher’s exact test was used. Linear regression was used to calculate associations between salivary rate and use of medication. The statistical software SPSS version 21.0 was used for statistical analyses.

## Results

As shown in Table 1, the study group was comparable with regard to age, within group gender distribution and Swedish or non-Swedish origin. The controls had a higher educational level and worked or studied full-time more often than the ASD group. Smoking and use of snuff were comparable in both groups. Medication and use of medication with reported side-effect hyposalivation was significantly more common in the ASD group compared to the control group.

The number of teeth was equal in both groups (27.4 ± 1.8 vs. 27.4 ± 1.4). Mean number of decayed, missing, or filled surfaces (DMFS) in adults diagnosed with ASD was 14.9 ± 18.9 compared to 15.9 ± 14.6 in the control group, a non-significant difference. We found no association between severity of ASD (reflected by higher AQ scores) and DMFS in the ASD group (*p* = 0.381).

Differences in the number of decayed surfaces (DS) and surfaces with initial caries lesions between the two groups were also non-significant. GBI% was lower in the ASD group 4.9 ± 6.2 (*n* = 46) compared to the controls 10.3 ± 17.2 (*n* = 58) (*p* = 0.046). The number of patients with buccal gingival recessions was significantly higher in the ASD group compared to the controls, 34/47 (72 %) versus 21/58 (36 %) (*p* < 0.001). Mean number of teeth with buccal gingival recessions was 6.3 ± 6.2 in the ASD group compared to 2.7 ± 4.8 in the control group (*p* = 0.001). Occurrence of calculus was equal in both groups (12/46, 26 % vs. 10/58, 17 %, *p* = 0.273).

The stimulated salivary secretion rate was 1.46 ± 0.72 in the ASD group (*n* = 43) compared to 2.74 ± 1.49 in the control group (*n* = 55) (*p* < 0.001). Only 6 persons (4 with ASD and 2 controls) had a salivary secretion rate below 0.7 ml/min. There was no correlation in the ASD group between number of medications and salivary secretion rate (*p* = 0.564). When comparing those with medication with reported side-effect hyposalivation (*n* = 21) to those without such medication (*n* = 22) in the ASD group, there were no significant differences regarding salivary secretion rate (1.34 ± 0.63 vs. 1.58 ± 0.80, *p* = 0.297). Un-medicated participants with ASD (*n* = 16) had a lower saliva secretion rate than in the control group (*n* = 51) (1.5 ± 0.7 and 2.7 ± 1.5, *p* = 0.048).

A large majority in both groups estimated their oral health as satisfactory. When comparing those with ASD, who estimated their oral heath to be good at the moment (*n* = 40) with those who did not (*n* = 7), there were significant differences regarding all caries variables. However, there were no differences in GBI% between those with ASD who estimated their oral heath as good and those who did not. When comparing those in the control group, who estimated their oral heath to be good with those who did not, there were significant differences regarding all caries variables and GBI%.

Table 2 presents the results of the oral health and dental hygiene habits questionnaire. Fewer patients in the ASD group brushed their teeth every morning (29/47, 62 %) compared to the control group (60/69, 87 %, *p* = 0.002). Table 3 presents the results of the questionnaire on dietary habits. Eating or drinking more that five times a day was answered positively by 49/69 (71 %) in the control group and 24/47 (51 %) in the ASD group (*p* = 0.029). Regarding previous dental experience there were no self-reported differences between the groups (Table 4). The most common reason for missed appointments was forgot to go in the ASD group (6/12, 50 %) and unable to go (due to illness, work, school or economical reasons) in the control group (8/12, 67 %).

**Table 2:** Questionnaire on oral health and dental hygiene habits in adults with autism spectrum disorder (*n* = 47) and a control group (*n* = 69)

Question	ASD group every day	Control group every day	*P* value^a^
Do you brush your teeth in the evening?	37/46 (81 %)	61/69 (88 %)	0.238
Do you brush your teeth in the morning?	29/47 (62 %)	60/69 (87 %)	0.002
Do you use dental floss?	8/46 (17 %)	8/69 (12 %)	0.379
Does your gingiva bleed when you brush your teeth?	1/47 (2 %)	3/69 (4 %)	0.646^b^
Do you use an electric toothbrush?	8/46 (17 %)	21/69 (30 %)	0.101

*ASD* Autism spectrum disorder

^a^Chi^2^ test

^b^Fisher’s exact test

**Table 3:** Dietary habits in adults with autism spectrum disorder (*n* = 47) and a control group (*n* = 69)

Question	ASD group yes	Control group yes	*P* value^a^
Do you eat or drink any food or beverage ≥5 times/d?	24/47 (51 %)	49/69 (71 %)	0.029
Do you eat mints, hard or chewy candies, candy bars, donuts, pastries, chips, crackers, or other similar snack foods between meals 3 days/week?	18/47 (38 %)	30/69 (43 %)	0.578
Do you drink non-diet soda, lemonade, fruit aids, sport drinks, or sugar (not sugar substitute)-sweetened tea or coffee between meals?	19/47 (40 %)	32/69 (46 %)	0.526

*ASD* Autism spectrum disorder

^a^Chi2-test

**Table 4:** Questions on previous dental experience in autism spectrum disorder (ASD) and control groups

Question	ASD	Control	*P* value^a^
Has visited the dentist in adulthood	46/47 (98 %)	66/68 (97 %)	0.758
Cancelled dental appointments during the past two years	17/44 (39 %)	30/68 (44 %)	0.566
Missed dental appointments during the past two years	13/44 (30 %)	14/68 (24 %)	0.249
Believes oral health at the moment is good	40/47 (85 %)	58/67 (87 %)	0.825

^a^Chi2-test

## Discussion

This study contributes to the understanding of oral health and oral health behaviours in patients with ASD. The major findings were that the caries prevalence in patients with ASD was similar to a matched control group, that the number of teeth with buccal gingival recessions was significantly higher and that the stimulated salivary flow rate was significantly lower in patients with ASD compared to controls.

As might be expected, the controls had a higher educational level and worked or studied full-time more often than the ASD group. Previous studies have shown that smoking among patients with ASD is rare [28], although psychiatric patients are significantly more often smokers than the general population. In this study, we found no significant differences in smoking prevalence between ASD and controls.

Conflicting results have been published regarding caries prevalence in patients with ASD. The results of this study show no significant difference in caries prevalence between ASD and controls. It is difficult to compare with previous studies, as our group of patients had no intellectual disabilities and were all living independently. Orellana et al. [12] studied adult patients with ASD and reported lower caries prevalence compared to controls. All patients presented with some degree of mental impairment and most of them were assisted with oral hygiene measures. The patients with ASD in the present study had an increased caries risk due to less frequent tooth brushing in the morning and lower salivary secretion rate but also protective factors such as less frequent snacking compared to controls. Patients with ASD had a significantly lower level of gingivitis compared to controls despite less frequent tooth brushing. Several factors can explain these findings; they had significantly more buccal surfaces with gingival recessions that indicate a persistent, intensive tooth brushing technique (possibly related to pervasive behaviours) and they also reported less frequent snacking, which generate lower levels of dental plaque.

The ASD group had a significantly lower salivary secretion rate compared to the control group, regardless of medication. Patients with ASD report more stress in everyday life [29] and they also report a higher level of dental anxiety [30], both factors that may negatively affect salivary secretion. Interestingly, the number of medications was not correlated to the salivary secretion rate. Previous studies in normal elderly patients on multi-drug regimens have reported the opposite finding [31]. Our finding, showing considerably lower saliva flow in adults with ASD compared to healthy controls, suggest that future studies should investigate whether saliva flow constitute a biological marker for ASD.

Self-perceived oral health was similar in both ASD and control groups. In the control group gingivitis was associated with self-perceived poor oral health, whereas this was not the case in the ASD group. It seems that only dental caries was of importance for the perception of good oral health among the participants with ASD. In other words the patients with ASD did not connect gingivitis to poor oral health.

During the course of our study, patients often expressed that receiving instructions on oral hygiene during the dental examination was confusing and hard to grasp, and that clinical instructions given after the examination would be more useful. Several of the patients in the ASD group expressed their gratitude regarding the written report on their dental status and the required treatments provided in this study. This suggests a need of individually adapted verbal and written instructions on oral heath status and oral hygiene (especially in order to avoid harmful tooth brushing) to individuals with ASD.

In contrast to previous studies [14, 15], the patients with ASD utilized the dental care equally compared to the controls. However, the result should be interpreted with caution, as the patients in the ASD group only consisted of those who had accepted the invitation to a dental examination, and the control group comprised typical adults with a regular dental contact. In a previous report on dental anxiety in the same patient group, it was found that 39/47 had booked their dental appointment due to reasons other than pain, such as regular check ups, indicating that the majority of the ASD group visited the dentist not only in emergency situations but on a regular basis [30]. It has been reported that patients with ASD have difficulties finding a dentist with necessary skills or willingness to treat them [15], but that particular study included a population of intellectual impaired patients, in contrast to the present study population.

Missed or cancelled appointments during the last two years were reported equally in both groups (ASD: 39 % vs. controls: 44 % and ASD: 30 % vs. controls: 24 %). These figures may be compared to 18-year-old Norwegians, of whom 10 % had cancelled and 23 % had missed at least one appointment during the course of one year [23]. In a study on 26 year olds in Scotland, 9.7 % of the study group had not visited a dentist in the previous 8 years and 41.8 % had booked their dental appointment because of a problem [32]. The most common self-reported reason for missed appointments in the ASD group was forgetfulness. Individuals with ASD typically display impaired executive function and have shown to have mild episodic memory impairments [33] that may contribute to this finding.

Some limitations should be mentioned. Several different dentists assessed the controls and the reliability between the dentists was not tested. On the other hand the same dentist examined all the patients’ X-rays. The dentist who examined the patients was not blinded to the condition of the patient; the use of one blinded dentist would have been preferable. Although poor dental health is consistently associated with severe mental illness in previous studies [34], dentists are presumably not considerably affected by the psychiatric condition of the patient in their assessment of the condition of the teeth.

The intriguing finding showing lower saliva flow in the patients with ASD than in controls could possibly be contributed to experiencing more stress during the appointment. Unfortunately we did not obtain any biomarkers for stress, such as cortisol levels or heart rate, in this study. Also, the feeling of stress in the ASD groups was limited by the cautious handling of the ASD patients in this study. Each one was given enough time for all their questions and the dentist chair was placed in the psychiatric clinic that was familiar to the patient.

Using self-reported data can be a limitation in the study, as bias may appear especially when questions are related to health behaviors, where social desirability can impact reporting. On the other hand there is no other convenient way to gather information on dental hygiene and dietary habits. Admitting poor dietary habits and poor grooming may cause shame, thus it is unlikely that acknowledgements of these behaviours would be greater in an interview than in a more anonymous self-report situation. Also, people with ASD are not prone to social desirability and they are able to respond to self-reports in a reliable way [35].

The controls were recruited from regular dental attendees as the majority of adults in Sweden. This group of patients have a better oral health and lower level of dental anxiety compared to non-attendees. Patients with particularly bad oral health or who value oral health less may have been less likely to participate in the study. This might have affected the results regarding caries prevalence and self perceived oral health. Another limitation is the relatively small sample size suggesting Type II error, for instance for the prevalence of smoking. Smoking was in fact more than three times more often reported by the controls compared to the ASD group. However, the significant differences between groups shown in this study cannot be contributed to large samples, which is a strength of our findings. Finally, the ASD group and the controls were not matched on educational level, as most people with ASD are unable to complete higher education. If the groups were matched on educational level our control group would perhaps have had poorer dental health than the ASD group. Instead, in our attempt to pursuit similar socioeconomic status between groups they were matched on place of residence, which is an indicator of socioeconomic background in Stockholm.

## Conclusions

Except for increased number of buccal gingival recessions, the oral health was comparable in adults with ASD and the control group. The patients with ASD did not connect gingivitis to poor oral health. The group with ASD had less snacking, but also less frequent tooth brushing in the mornings. The stimulated saliva secretion was lower in the ASD group, regardless of medication. The contacts with dental care were equal between the ASD and the control group. The most common reported reason for missing a dental appointment was forgetfulness in the ASD group. Despite equal caries prevalence, the risk for reduced oral health due to decreased salivary flow should be taken into consideration when planning dental care for patients with ASD. We suggest the use of written reminders of dental appointments and written and verbal report on oral health status and oral hygiene instructions.

## Abbreviations

ASD: Autism spectrum disorder
AQ: Autism-spectrum quotient
DMFS: Decayed missing filled surfaces
DS: Decayed surfaces
DSM-IV-TR: Diagnostic and statistical manual of mental disorder, 4^th^ edition, text revised
DSM5: Diagnostic and statistical manual of mental disorder, 5^th^ edition
GBI: Gingival bleeding index
PDD-NOS: Pervasive developmental disorder, not otherwise specified
WAIS-III-R: Wechsler adult intelligence scale, revised

## References

[1] Russel G, Rodger LR, Ukommune OC, Ford T (2014). Prevalence of Parent-Reported ASD and ADHD in the UK: Findings from the Millennium Cohort Study. J Autism Dev Disord.

[2] American Psychiatric Association: Diagnostic and Statistical Manual of Mental Disorder, DSM5. Washington D.C.; 2013.

[3] American Psychiatric Association: Diagnostic and Statistical Manual of Mental Disorder, DSM-IV-TR. Washington D.C.; 2000.

[4] Gomot M, Wicker B (2012). A challenging, unpredictable world for people with Autism Spectrum Disorder. Int J Psychophysiol.

[5] Klintwall L, Holm A, Eriksson M, Carlsson LH, Olsson MB, Hedvall A, Gillberg C, Fernell E (2011). Sensory abnormalities in autism – a brief report. Res Dev Disabil.

[6] Markram H, Rinaldi T, Markram K (2007). The intense world syndrome-an alternative hypothesis for autism. Front Neurosci.

[7] Stein LI, Polido JC, Maillaux Z, Coleman GG, Cermak SA (2011). Oral care and sensory sensitivities in children with autism spectrum disorder. Spec Care Dentist.

[8] Kopycka-Kedzierawski DT, Auinger P (2008). Dental needs and status of autistic children: results from the National Survey of Children's Health. Pediatr Dent.

[9] Bassaoukou IH, Nicolau J, Dos Santos MT (2009). Saliva flow rate, buffer capacity, and pH of autistic individuals. Clin Oral Investig.

[10] Rai K, Hedge AM, Jose N (2012). Salivary antioxidants and oral health in children with autism. Arch Oral Biol.

[11] Loo CY, Graham RM, Hughes CV (2008). The caries experience and behaviour of dental patients with autism spectrum disorder. JADA.

[12] Orellana LM, Silvestre FJ, Martinez-Sanchis S, Martinez-Mihi V, Bautista D (2012). Oral manifestations in a group of adults with autism spectrum disorder. Med Oral Patol Oral Bucal.

[13] Hsia Y, Wong AY, Murphy DG, Simonoff E, Buitelaar JK, Wong IC (2014). Psychopharmacological prescriptions for people with autism spectrum disorder (ASD): a multinational study. Psychopharmacology.

[14] Lai B, Milano M, Roberts MW, Hooper SR (2012). Unmet dental needs and barriers to dental care among children with autism spectrum disorders. J Autism Dev Disord.

[15] Brickhouse TH, Farrington FH, Best AM, Ellisworth CW (2009). Barriers to dental care for children in Virginia with autism spectrum disorders. J Dent Child.

[16] Baron-Cohen S, Wheelright S, Skinner R, Martin J, Clubley E (2001). The autism-spectrum quotient (AQ): evidence from Asperger syndrome/high-functioning autism, males and females, scientists and mathematicians. J Autism Dev Disord.

[17] Ruzich E, Allison C, Smith P, Watson P, Auyeung B, Ring H, Baron-Cohen S (2015). Measuring autistic traits in the general population: a systematic review of the Autism-Spectrum Quotient (AQ) in a nonclinical population sample of 6,900 typical adult males and females. Mol Autism.

[18] Bishop SL, Seltzer MM (2012). Self-reported autism symptoms in adults with Autism Spectrum Disorders. J Autism Dev Disord.

[19] Ring H, Woodbury-Smith M, Watson P, Wheelwright S, Baron-Cohen S (2008). Clinical heterogeneity among people with high functioning autism spectrum conditions: evidence favouring a continuous severity gradient. Behav Brain Funct.

[20] Hollender L, Koch G (1969). Influence of topical application of fluoride on rate of progress of carious lesions in children. A long-term roentgenographic follow-up. Odontol Revy.

[21] Shwartz M, Gröndahl HG, Pliskin JS, Boffa J (1984). A longitudinal analysis from bite-wing radiographs of the rate of progression of approximal carious lesions through human dental enamel. Arch Oral Biol.

[22] Axelsson P, Linde J (1975). Effect of fluoride on gingivitis and dental caries in a preventive program based on plaque control. Community Dent Oral Epidemiol.

[23] Skaret E, Raadal M, Berg E, Kvale G (1998). Missed and cancelled appointments among 12–18 year-olds in the Norwegian Public Dental Service. Eur J Oral Sci.

[24] Julihn A, Barr Agholme M, Grindefjord M, Modeer T (2006). Risk factors and risk indicators associated with high caries experience in Swedish 19-year-olds. Acta Odontol Scand.

[25] Blomqvist M, Holmberg K, Fernell E, Ek U, Dahllöf G (2007). Dental caries and oral health behavior in children with attention deficit hyperactivity disorder. Eur J Oral Sci.

[26] Blomqvist M, Ahadi S, Fernell E, Ek U, Dahllöf G (2011). Dental caries in adolescents with attention deficit hyperactivity disorder: a population-based follow-up study. Eur J Oral Sci.

[27] Mobley CC (2003). Nutrition and dental caries. Dent Clin North Am.

[28] Bejerot S, Nylander L (2003). Low prevalence of smoking in patients with autism spectrum disorders. Psychiatry Res.

[29] Hirvikoski T, Blomqvist M (2014). High self-perceived stress and poor coping in intellectually able adults with autism spectrum disorder. Autism.

[30] Blomqvist M, Dahllöf G, Bejerot S (2014). Experience of dental care and dental anxiety in adults with autism spectrum disorder. Autism Res Treat.

[31] Nederfors T (2000). Xerostomia and Hyposalivation. Adv Dent Res.

[32] Thomson WM, Locker D, Poulton R (2000). Incidence of dental anxiety in young adults in relation to dental treatment experience. Community Dent Oral Epidemiol.

[33] Meyer BJ, Gardiner JM, Bowler DM (2014). Directed forgetting in High-Functioning Adults with Autism Spectrum Disorders. J Autism Dev Disord.

[34] Kisely S, Quek LH, Pais J, Lalloo R, Johnson NW, Lawrence D (2011). Advanced dental disease in people with severe mental illness: systematic review and meta-analysis. Br J Psychiatry.

[35] Hesselmark E, Eriksson JM, Westerlund J, Bejerot S (2015). Autism Spectrum Disorders and Self-reports: Testing Validity and Reliability Using the NEO-PI-R. J Autism Dev Disord.

